# Supplementary material for: Evaluation of Matrix-Assisted Laser Desorption Ionization–Time of Flight Mass Spectrometry for Molecular Typing of Acinetobacter baumannii in Comparison with Orthogonal Methods
Source: Microbiol Spectr. 2023 May 8;11(3):e04995-22. doi: 10.1128/spectrum.04995-22 (PMC10269802; doi:10.1128/spectrum.04995-22)

Evaluation of MALDI-TOF MS for molecular typing of *Acinetobacter baumannii* in comparison with orthogonal methods

SUPPLEMENTARY INFORMATION

Eloise J Busby[1], Ronan M Doyle[2,3], Clara Leboreiro Babe*[4], Kathryn A Harris[5], Damien Mack[4,6], Gema Méndez-Cervantes[7], Denise M O'Sullivan[1], Vicky Pang[6], Zahra Sadouki[4], Priya Solanki*[4], Jim F Huggett[1,8], Timothy D McHugh[4], Emmanuel Q Wey✉[4,6]

* These authors contributed equally to this work

✉ Corresponding author Emmanuel.wey@nhs.net

[1]National Measurement Laboratory, LGC, Queens Road, Teddington, Middlesex, UK

[2]Department of Microbiology, Virology and Infection Control, Great Ormond Street Hospital for Children NHS Foundation Trust, London, UK

[3]Clinical Research Department, London School of Hygiene & Tropical Medicine, London, UK

[4]Centre for Clinical Microbiology, Royal Free Campus, Division of Infection and Immunity, Faculty of Medical Sciences, University College London, UK

[5]Virology Department, ESEL Pathology Partnership, Royal London Hospital, Barts Health NHS Trust, London, UK

[6]Royal Free Hospital NHS Foundation Trust, London, UK

[7]Clover Bioanalytical Software, SL. Granada, Spain

[8]School of Biosciences & Medicine, Faculty of Health & Medical Science, University of Surrey, Guildford, UK

Table/figure legends

Supplementary file S1 ('.xlsx'): Whole genome sequencing (WGS) single nucleotide variant (SNV) matrix for the 31 *A. baumannii* isolates. Isolates in red are the three HCAI outbreak associated isolates identified as being part of a unique transmission event. Isolates in yellow were part of the INHALE study cohort and include an isolate (Abau-Iso2-11) that contained few SNV differences compared to the HCAI isolates in red.

Supplementary file S2 ('.png'): Heat map showing SNV differences between isolates. Cut-off was set to 15 base changes; 0-15: Red, 15-Max: Orange to blue.

Supplementary file S3 ('.xlsx'): MALDI-TOF MS 'Bruker FlexAnalysis peak data' ('.xlsx' files derived from processed '.mzXML' files).

Table S1: Reference laboratory typing results for pulsed-field gel electrophoresis (PFGE) and variable nucleotide tandem repeat (VNTR) profiles (where available) for the 31 *A. baumannii* isolates from the Royal Free London NHS Foundation Trust. The 18 HCAI outbreak associated isolates are pre-fixed with 'MBT16' and the 13 additional reference isolates are denoted 'Abau-Iso2'.

Table S2: MALDI-TOF MS analysis of 18 HCAI outbreak associated *A. baumannii* isolates (a) MALDI-TOF MS peak classes identified as potential biomarkers for strain typing using the Bruker FlexAnalysis method. Black represents presence of peak, blank represents absence. (b) Bruker MALDI Biotyper groups based on presence or absence of visually chosen mass to charge (m/z) peak classes illustrated in Table 2a.

Table S3: UniProtKB/Swiss-Prot search results for *A. baumannii* (Tax ID: 470) performed using TagIdent tool (https://web.expasy.org/tagident/ accessed 06/05/2022). MW range for the search was 2 to 9 kDa. Possible matched proteins (Da) and m/z peaks for each MALDI-TOF MS data analysis approach used for the 18 HCAI outbreak associated isolates are highlighted in green.

Figure S1: Hierarchal clustering of the 18 HCAI outbreak associated *A. baumannii* isolates calculated in Clover MS data analysis software using UPGMA based on MALDI-TOF MS spectra. The isolates clustered into two main groups; Group I and Group II.

Figure S2: Epidemiological data were collected for the 18 HCAI outbreak associated isolates. (a) Timeline of patient migration within ward A and other hospital wards. Patient ID is given along with isolate number. The red box highlights the time point in which Patients 2 & 4 crossed on ward A; green arrows indicate approximate date of first MDR A. baumannii isolation. (b) Floor map of Ward A. The associated 4-bedded male ward is highlighted by a yellow box, with relevant beds for patients 2 & 4 highlighted by black stars.

Table S1

Study ID	Year isolated	PFGE result	VNTR profile	MALDI Biotype	FTIR cluster	Patient ID (HCAI[†] outbreak)	Additional information
MBT16-003	2014	OXA-23 clone 1	10,20,12, 6	A	Cluster_331	014	Matched[‡] to MBT16-042 (Patient 013)
MBT16-005§	2015	OXA-23 clone 1	9,20,10, 6	B	Cluster_331	002*	Matched to MBT16-039, -011 (Patient 002, 004)
MBT16-008	2014	OXA-23 clone 1	9,20,11, 6	A	Cluster_328	010	
MBT16-011§	2015	OXA-23 clone 1	9,20,10, 6	B	Cluster_331	004	Matched to MBT16-008 (Patient 010)
MBT16-015	2015	OXA-23 clone 1	-,20,12, 6	G	Cluster_328	005	
MBT16-016	2014	OXA-23 clone 1	10,20,12, 6	C	Cluster_328	012	Matched to MBT16-042, -003, -018, -059

							(Patient 013, 014, 015, 016)
MBT16-018	2014	OXA-23 clone 1	10,20,12, 6	D	Cluster_328	015	Matched to MBT16-003 (Patient 014) & MBT16-042 (Patient 013)
MBT16-025	2015	OXA-23 clone 1	10,20,12, 7	C	Cluster_328	003**	Matched to MBT16-030 (Patient 007)
MBT16-029	2015	OXA-23 clone 1	10,20,12, 6	E	Cluster_323	006	Matched to MBT16-033 (Patient 011)
MBT16-030	2015	OXA-23 clone 1	10,20,12, 6	H	Cluster_323	007	Matched to MBT16-029 (Patient 006)
MBT16-031	2015	OXA-23 clone 1	10,20,13, 6	D	Cluster_328	003**	Matched to MBT16-030 (Patient 007)
MBT16-033	2014	OXA-23 clone 1	10,20,12, 6	F	Cluster_328	011	Matched to MBT16-016 (Patient 012)

MBT16-039§	2015	OXA-23 clone 1	9,20,10, 6	B	Cluster_331	002*	Matched to MBT16-005, -011 (Patient 002, 004)
MBT16-040	2015	OXA-23 clone 1	10,20,12, 6	F	Cluster_328	001	Matched to MBT16-031 & MBT16-025 (Patient 003)
MBT16-042	2014	OXA-23 clone 1	10,20,12, 6	C	Cluster_328	013	
MBT16-059	2014	OXA-23 clone 1	10,20,12, 6	E	Cluster_328	016	Matched to MBT16-003, -018, -042 (Patient 014, 015, 013)
MBT16-060	2015	OXA-23 clone 1	10,20,12, 6	C	Cluster_328	006	Matched to MBT16-033 (Patient 011)
MBT16-062	2015			C	Cluster_323	009	Not sent to reference laboratory
Abau-Iso2-03	2016			NA	Cluster_332	NA	OXA-23-like bla positive

Abau-Iso2-05	2016			NA	Cluster_271	NA	bla OXA-51 (intrinsic)-like positive; OXA-58, OXA-51 and OXA-23 negative; NDM positive; VIM/SIM/GIM/SPM/IMP negative
Abau-Iso2-11	2017			NA	Cluster_307	NA	bla OXA-58 and -40-like negative; OXA-23 and 51-like positive; fully resistant to everything, except colistin and co-trimoxazole
Abau-Iso2-15	2011	Unique	24, 17	NA	Cluster_232	NA	bla OXA-23-like positive; bla OXA51-like positive

Abau-Iso2-16	2012	Unique	NA	Cluster_272	NA	bla OXA-58-like negative; bla OXA-23-like positive; bla OXA-51-like positive; bla OXA-40-like negative; Class 1 integrase gene positive
Abau-Iso2-19	2017		NA	Cluster_333	NA	Fully resistant to everything except colistin; OXA-23 positive
Abau-Iso2-20	2017		NA	Cluster_333	NA	Susceptible to amikacin and gentamicin, resistant to meropenem, co-trimoxazole and ciprofloxacin; OXA-23 positive

Abau-Iso2-23	2016			NA	Cluster_333	NA	OXA-23, OXA-51 positive
Abau-Iso2-24	2016			NA	Cluster_329	NA	OXA-23 blaNIM positive
Abau-Iso2-25	2016			NA	Cluster_332	NA	bla OXA-51 (intrinsic)-like positive; OXA58, OXA 51 and OXA23 negative; NDM positive; VIM/SIM/GIM/SPM/IMP all negative
Abau-Iso2-26	2016	International Clone II lineage		NA	Cluster_333	NA	OXA-23 OXA-51 positive
Abau-Iso2-27	2016	International Clone II lineage; PFGE Result: RFRE04AC-10	26,25,-,10	NA	Cluster_290	NA	blaOXA-58-like negative; blaOXA-23-like negative; blaOXA-51-like positive; blaOXA-40-like negative;

						Class 1 integrase gene positive. Identification was by detection of the bla (OXA-51-like) carbapenemase gene intrinsic in *A.baumannii*, expression of which is dependent on provision of a promoter by an insertion sequence element
Abau-Iso2-28	2016		NA	Cluster_329	NA	OXA-23 blaNIM positive

†HCAI – Healthcare associated infection. ‡ Matching VNTR and/or PFGE profiles. § MBT16-005 contained 1 SNV difference compared to MBT16-011 and MBT16-039. The latter two isolates contained 2 SNV differences compared to one another. NA – not applicable. Asterisks indicate where isolates were derived from the same patient(s).

Table S2

(a)	Peak class (m/z)			
**Isolate**	**2256**	**2585**	**5434**	**5448**
MBT16-003			■	
MBT16-005		■		
MBT16-008			■	
MBT16-011		■		
MBT16-015				
MBT16-016	■		■	
MBT16-018		■	■	
MBT16-025	■		■	
MBT16-029	■	■	■	
MBT16-030	■	■	■	■
MBT16-031		■	■	
MBT16-033	■		■	■
MBT16-039		■		
MBT16-040	■		■	■
MBT16-042	■		■	
MBT16-059	■	■	■	
MBT16-060	■		■	
MBT16-062	■		■	

(b)	MALDI Biotype	Isolate
	A	MBT16-003
	A	MBT16-008
	B	MBT16-005
	B	MBT16-011
	B	MBT16-039
	C	MBT16-016
	C	MBT16-025
	C	MBT16-042
	C	MBT16-060
	C	MBT16-062
	D	MBT16-018
	D	MBT16-031
	E	MBT16-029
	E	MBT16-059
	F	MBT16-033
	F	MBT16-040
	G	MBT16-015
	H	MBT16-030

Figure S1

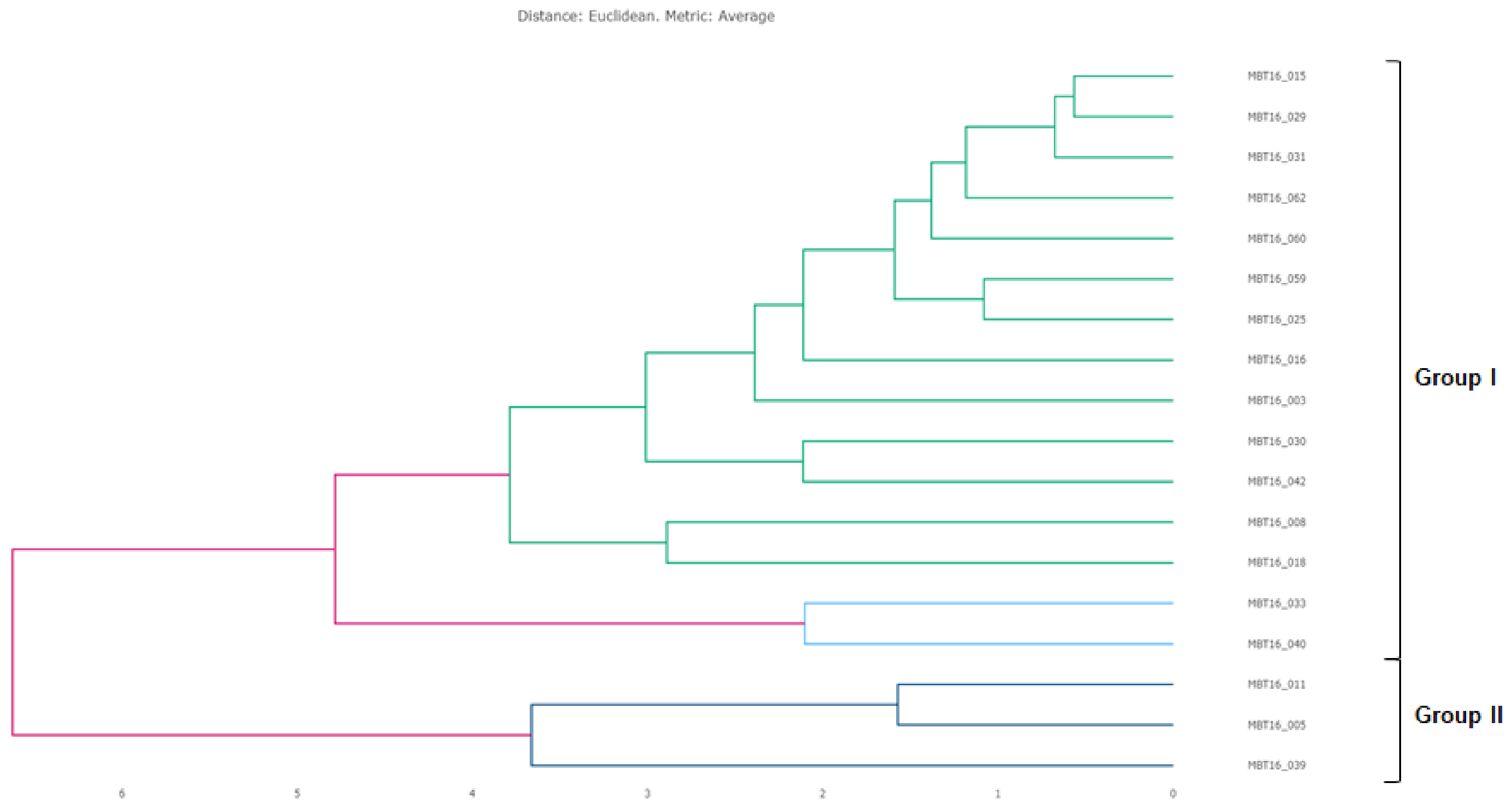
Distance: Euclidean. Metric: Average
MBT16_015
MBT16_029
MBT16_031
MBT16_062
MBT16_060
MBT16_059
MBT16_025
MBT16_016
MBT16_003
MBT16_030
MBT16_042
MBT16_008
MBT16_018
MBT16_033
MBT16_040
MBT16_011
MBT16_005
MBT16_039
6
5
4
3
2
1
0
Group I
Group II

Table S3

MW (Da)	Protein	BioNumerics	Clover MS Data Analysis Software
2805	Aspartate 1-decarboxylase beta chain.	2150	2256
2938	Coenzyme PQQ synthesis protein A.	2873	2585
3980	Phosphatidylserine decarboxylase alpha chain. {ECO:000025...	3073	3073
3996	Phosphatidylserine decarboxylase alpha chain. {ECO:000025...	3317	3723
4265	50S ribosomal protein L36.	3338	4245
5175	50S ribosomal protein L34.	3444	5178
5189	50S ribosomal protein L34.	3723	5434
5462	UPF0391 membrane protein A1S_3910.	4245	5448
6090	50S ribosomal protein L33.	4257	5751
6642	50S ribosomal protein L30.	4267	5771
7080	50S ribosomal protein L32.	4492	8487
7402	50S ribosomal protein L35.	5034	
7435	50S ribosomal protein L29.	5178	
7719	Sec-independent protein translocase protein TatA.	5434	
8360	ATP synthase subunit c.	5751	
8451	30S ribosomal protein S21.	5772	
8492	Translation initiation factor IF-1.	6094	
8669	Acyl carrier protein.	6330	

8992	30S ribosomal protein S18.	7439
		8490
		8723
		8984

Figure S2

(a)

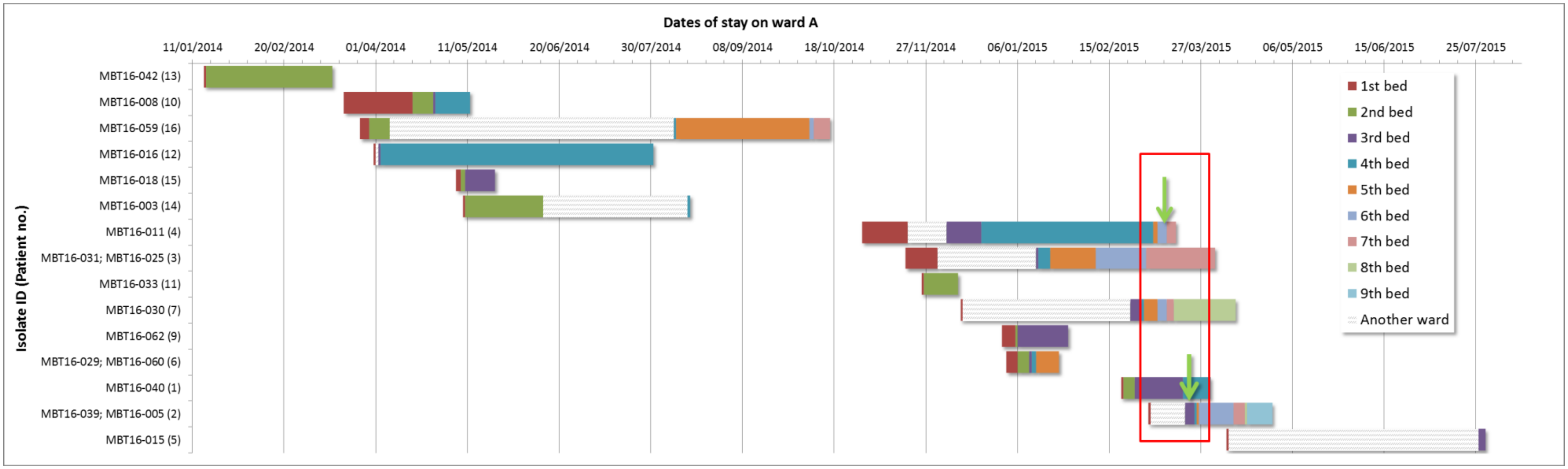

Dates of stay on ward A
11/01/2014
20/02/2014
01/04/2014
11/05/2014
20/06/2014
30/07/2014
08/09/2014
18/10/2014
27/11/2014
06/01/2015
15/02/2015
27/03/2015
06/05/2015
15/06/2015
25/07/2015
Isolate ID (Patient no.)
MBT16-042 (13)
MBT16-008 (10)
MBT16-059 (16)
MBT16-016 (12)
MBT16-018 (15)
MBT16-003 (14)
MBT16-011 (4)
MBT16-031; MBT16-025 (3)
MBT16-033 (11)
MBT16-030 (7)
MBT16-062 (9)
MBT16-029; MBT16-060 (6)
MBT16-040 (1)
MBT16-039; MBT16-005 (2)
MBT16-015 (5)
1st bed
2nd bed
3rd bed
4th bed
5th bed
6th bed
7th bed
8th bed
9th bed
Another ward

(b)

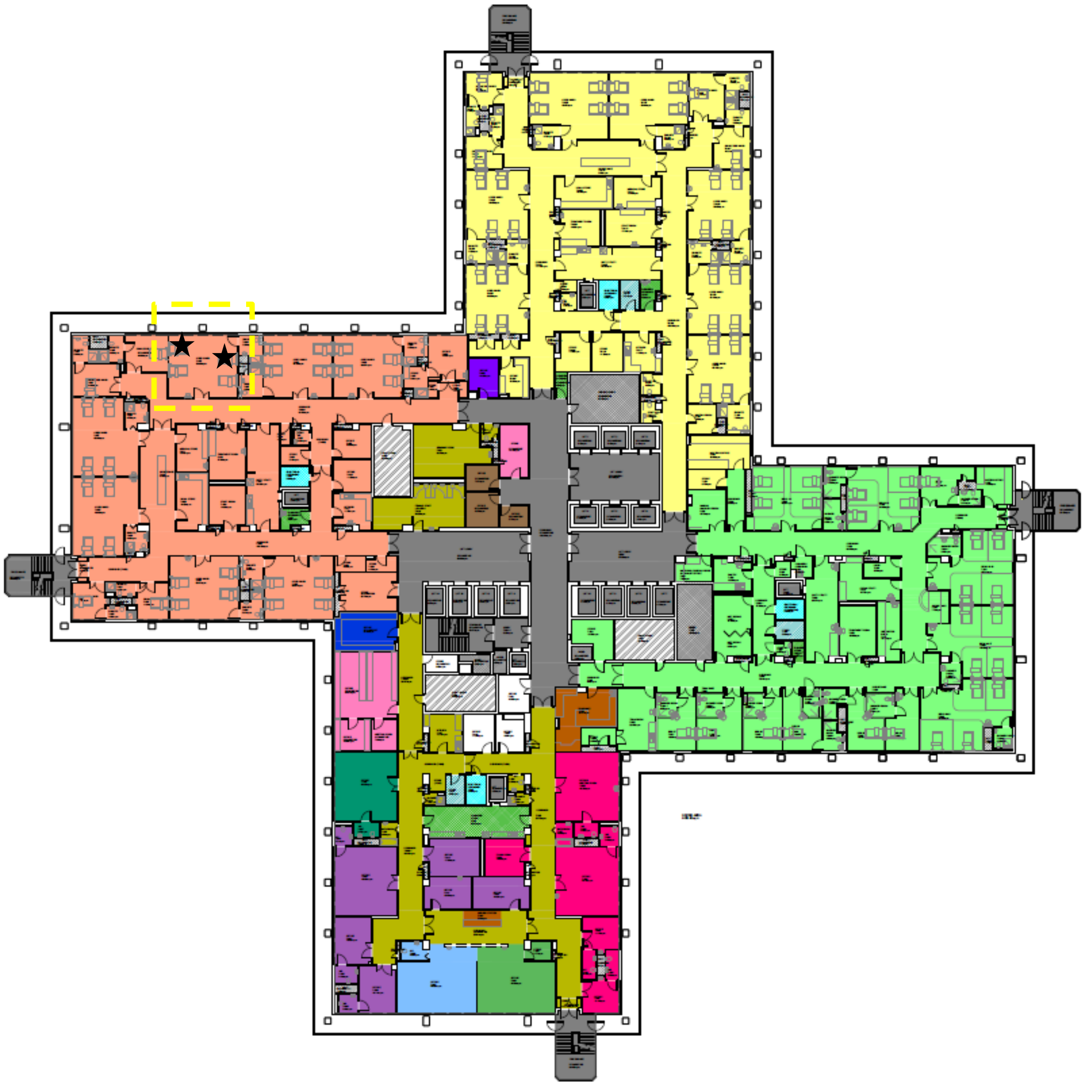

Supplement: Supplemental file 4 — Tables S1 to S3 and Fig. S1 and S2. Download spectrum.04995-22-s0001.pdf, PDF file, 0.6 MB [file spectrum.04995-22-s0001.pdf]
