# Supplementary figures and images for: Evaluation of Matrix-Assisted Laser Desorption Ionization–Time of Flight Mass Spectrometry for Molecular Typing of Acinetobacter baumannii in Comparison with Orthogonal Methods
Source: Microbiol Spectr. 2023 May 8;11(3):e04995-22. doi: 10.1128/spectrum.04995-22 (PMC10269802; doi:10.1128/spectrum.04995-22)

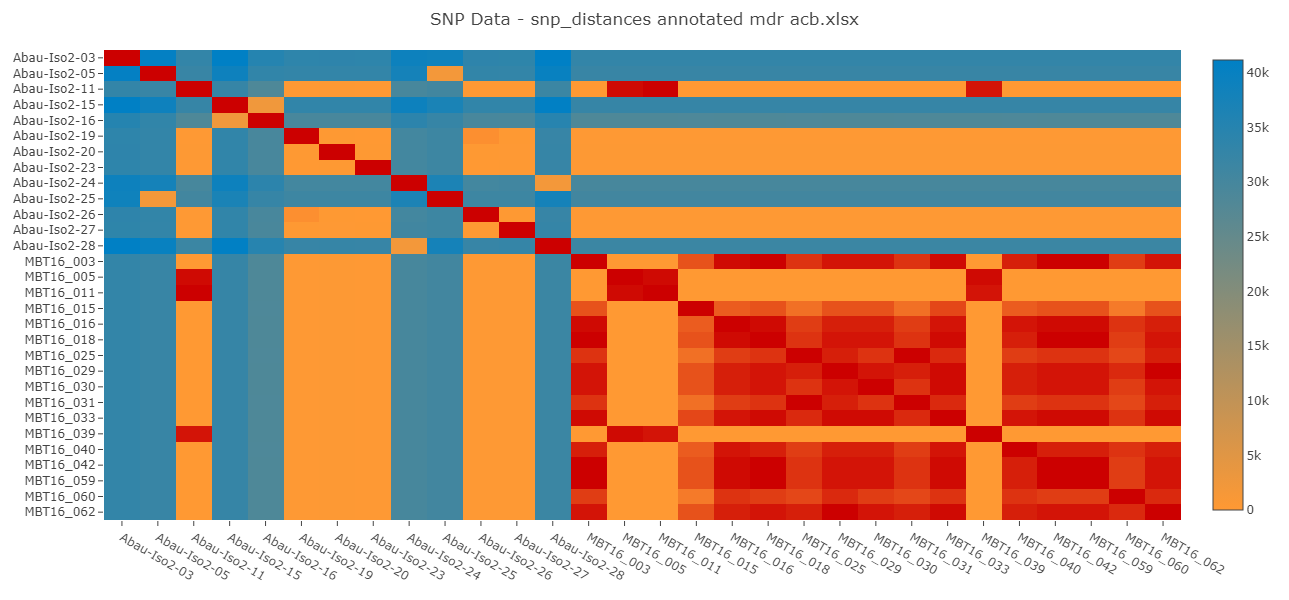

Supplement: Supplemental file 2 — Heatmap of SNV differences. Download spectrum.04995-22-s0003.png, PNG file, 0.08 MB [file spectrum.04995-22-s0003.png]
